# Supplementary material for: A stable quasi-solid electrolyte improves the safe operation of highly efficient lithium-metal pouch cells in harsh environments
Source: Nat Commun. 2022 Mar 21;13:1510. doi: 10.1038/s41467-022-29118-6 (PMC8938510; doi:10.1038/s41467-022-29118-6)
Supplement: Supplementary file 1 — Supplementary Information [file 41467_2022_29118_MOESM1_ESM.pdf]

Supplementary Information for

A stable quasi-solid electrolyte boosts the safe operation of high-efficiency lithium-metal pouch-cell in harsh environment

Authors: Zhi Chang[1], Huijun Yang[1,2], Xingyu Zhu[1,2], Ping He[3] and Haoshen Zhou[1,2,3]*

Affiliations:

[1]Energy Technology Research Institute, National Institute of Advanced Industrial Science and Technology (AIST), 1-1-1, Umezono, Tsukuba 305-8568, Japan.

[2]Graduate School of System and Information Engineering, University of Tsukuba, 1-1-1, Tennoudai, Tsukuba 305-8573, Japan.

[3]Center of Energy Storage Materials & Technology, College of Engineering and Applied Sciences, Jiangsu Key Laboratory of Artificial Functional Materials, National Laboratory of Solid State Micro-structures, and Collaborative Innovation Center of Advanced Micro-structures, Nanjing University, Nanjing 210093, P. R. China.

*Correspondence to: hszhou@nju.edu.cn.

Supplementary Discussion

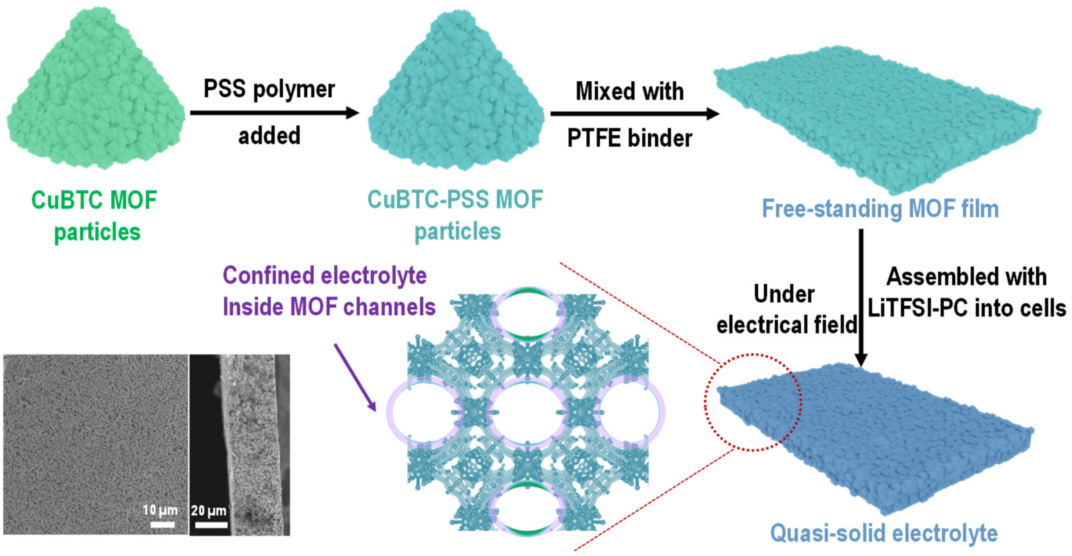

Figure S1. The schematic illustration for the preparation process of the quasi-solid electrolyte.

The afore-prepared MOF composites were then physically mixed with polytetrafluoroethylene (PTFE) to prepare flexible MOF films. Noting that before assembled into cells, the obtained MOF films were under a vacuumed heat-treatment to activate the MOF films (vacuum heated at 180 °C to remove moisture within MOF cavities).[1] The activated MOF can facilitate the infiltration of electrolyte.

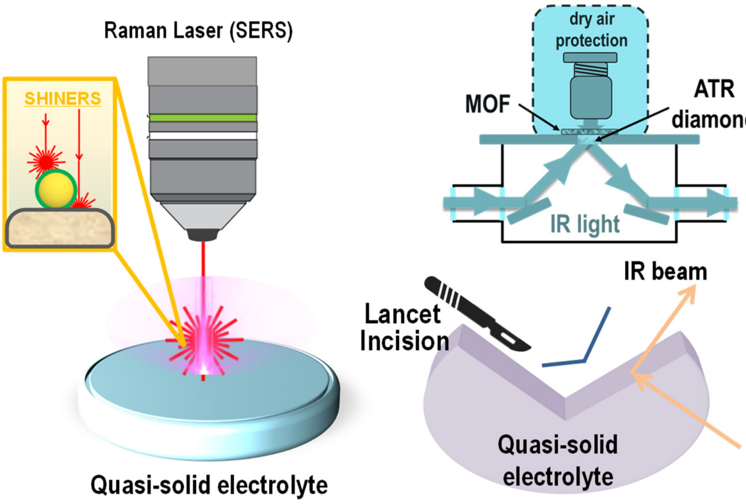

Figure S2. Schematic illustration of the operando-Raman and ATR-FTIR characterization system employed in this study.

Typically, the scattering signal used in common Raman spectroscopy was relatively weak, therefore, in certain circumstances, for certain samples, it is extremely hard to collect useful signals. To collecting strong and clear peaks, a SHINERS (shell-isolated nanoparticle-enhanced Raman spectroscopy) technique that greatly enhances the intensity of scattering signal was adopted.[2] Points from MOF were selected for the operando-Raman and ATR-FTIR test to study the configuration of electrolytes confined inside MOF channels.

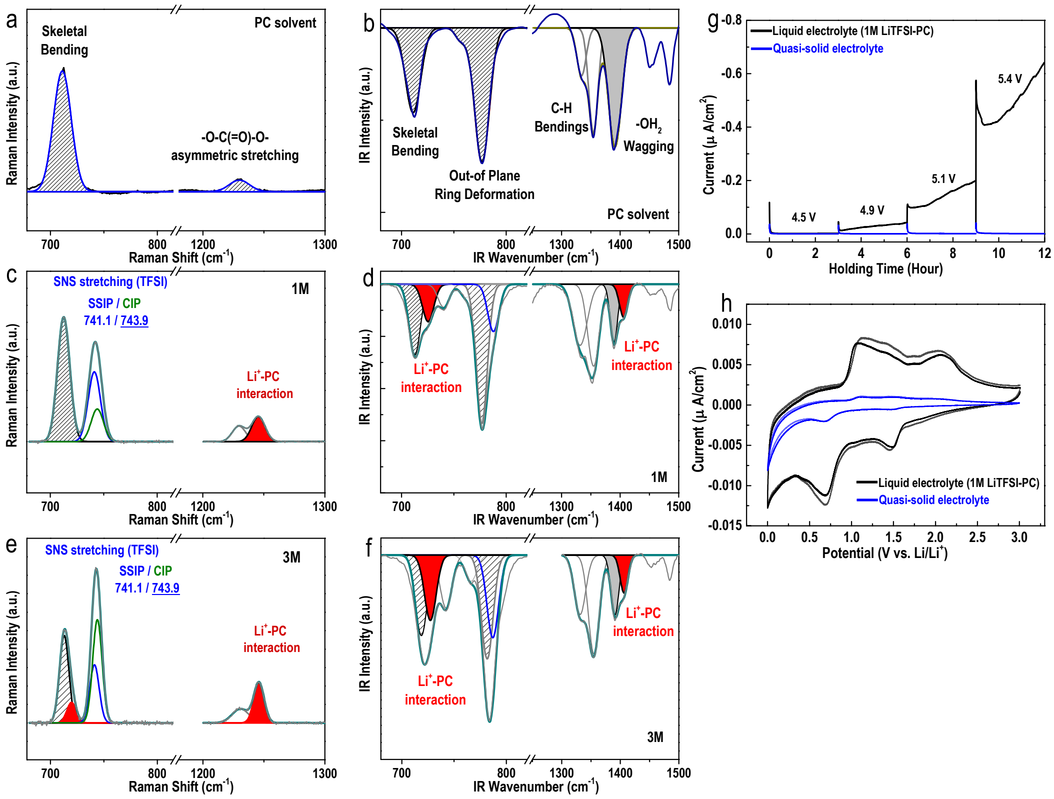

Figure S3. Raman and ATR-FTIR spectra of (a, b) PC solvent, (c, d) 1M typical liquid and (e, f) concentrated electrolyte. (g) Potentiostatic Intermittent Titration Technique floating test and (h) Cyclic voltammograms of typical liquid electrolyte and the prepared quasi-solid electrolyte.

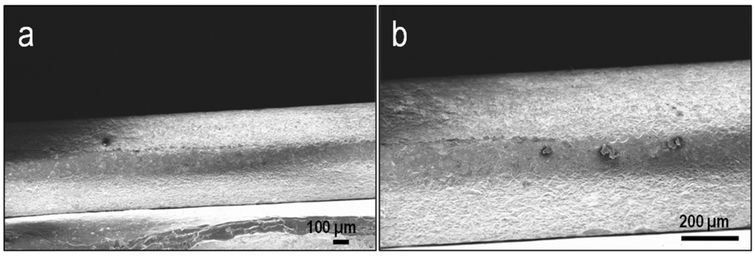

Figure S4. SEM images of the commercial $Li_{1.3}Al_{0.3}Ge_{1.7}(PO_4)_3$ (LAGP) solid electrolyte).

According to the SEM images, the commercial LAGP solid electrolyte is much thick than our prepared quasi-solid electrolyte.

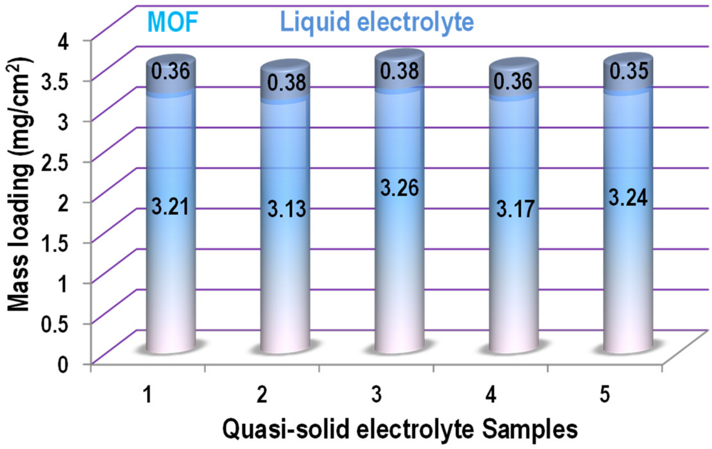

Figure S5. The weights of the five quasi-solid electrolytes.

To test the exact amount of liquid electrolyte confined inside MOF channels, five MOF film samples before and after electrochemical cycling processes within LiTFSI-PC electrolyte were employed. The average amount of liquid electrolyte confined was calculated to be about 9% among the whole weigh of the final obtained quasi-solid electrolytes. Given the density of conventional 1M PC-LiTFSI carbonate electrolyte (100 μL electrolyte weights 129.5 mg cm^{-2}), which means only 0.23 μL cm^{-2} (corresponding to 0.3 mg cm^{-2}) was contained in our prepared quasi-solid electrolyte.

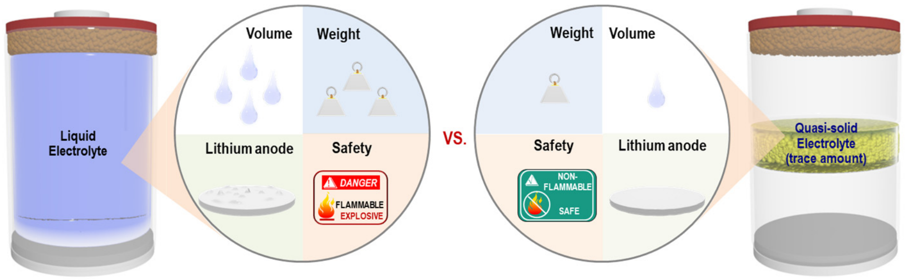

Figure S6. Schematical illustration for the advantages of the prepared quasi-solid electrolyte in constructing highly efficient lithium-metal batteries.

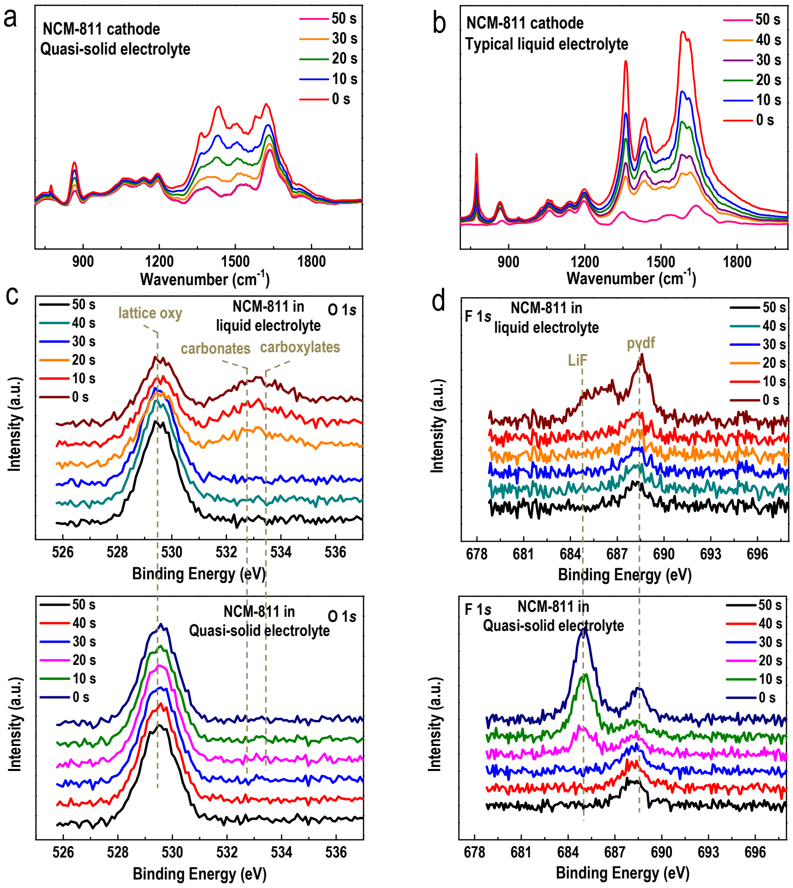

Figure S7. Selected etching FT-IR spectra recorded from the cycled NCM-811 cathodes cycled within (a) quasi-solid electrolyte and (b) typical liquid electrolyte. (c) O 1*s* and (d) F 1*s* of selected etching XPS spectra recorded from cycled NMC-811 cycled within quasi-solid electrolyte and typical liquid electrolyte. (top: NMC-811 typical liquid electrolyte; bottom: NMC-811 cycled within quasi-solid electrolyte).

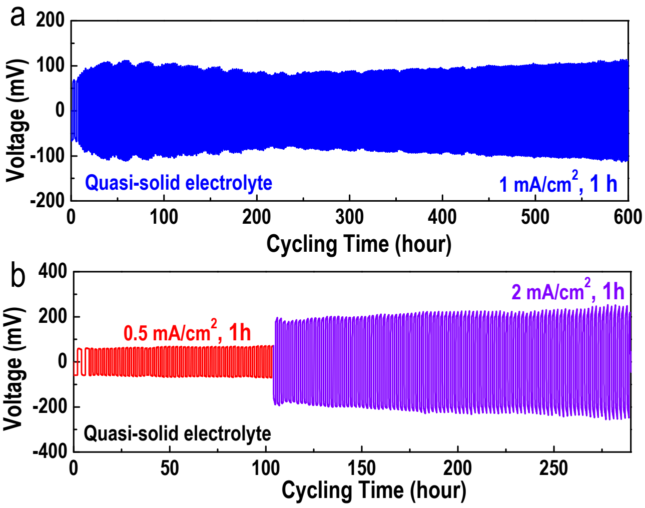

Figure S8. Compatibility of the quasi-solid electrolyte towards lithium metal: Electrochemical performances of Li//Li symmetrical cells used quasi-solid electrolyte. (a) Li//Li cells under 1 mA cm^{-2}, 1 mAh cm^{-2}. (b) Li//Li cells tested under 0.5 mA cm^{-2}, 0.5 mAh cm^{-2} and 2 mA cm^{-2}, 2 mAh cm^{-2}.

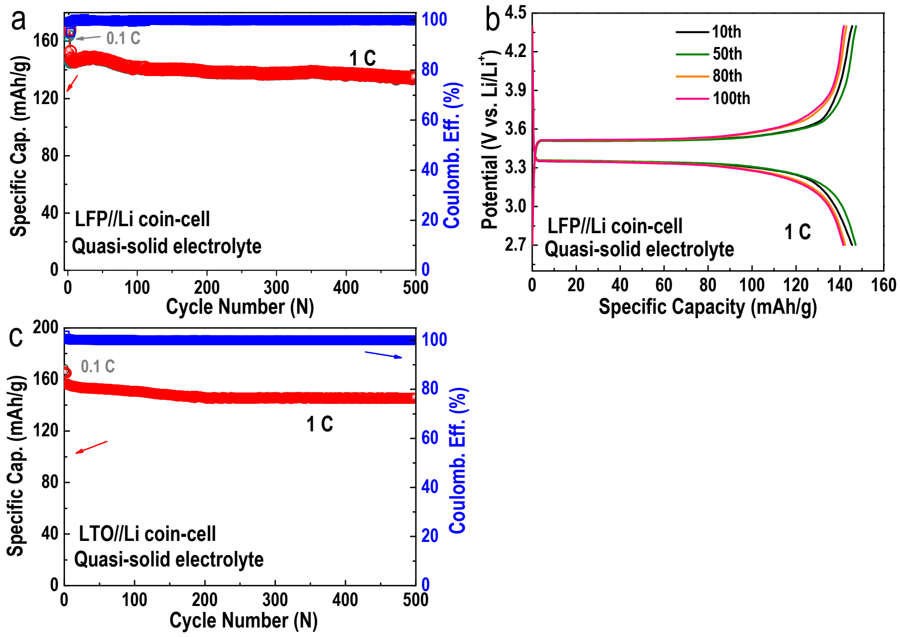

Figure S9. Electrochemical performances of LFP//Li and LTO//Li coin-cells used quasi-solid electrolyte. (a) cycling performance (LFP//Li) and the corresponding (b) discharge/charge curves of the $LiFePO_4$//Li (LFP//Li) coin-cell used quasi-solid electrolyte. (c) cycling performance $Li_4Ti_5O_{12}$//Li (LTO//Li) coin-cell assembled with quasi-solid electrolyte.

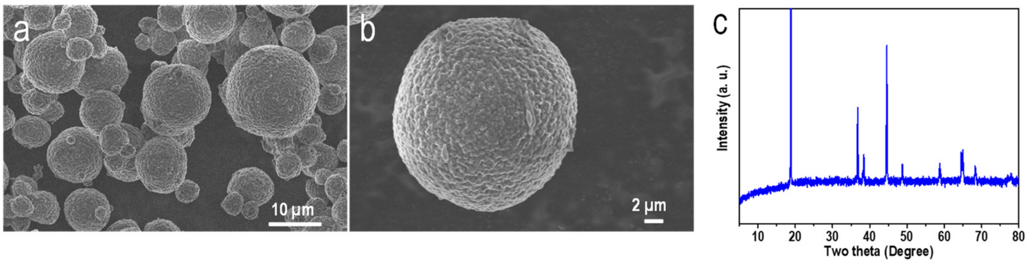

Figure S10. (a, b) SEM images and (c) XRD pattern of the pristine NCM-811 cathode material powder.

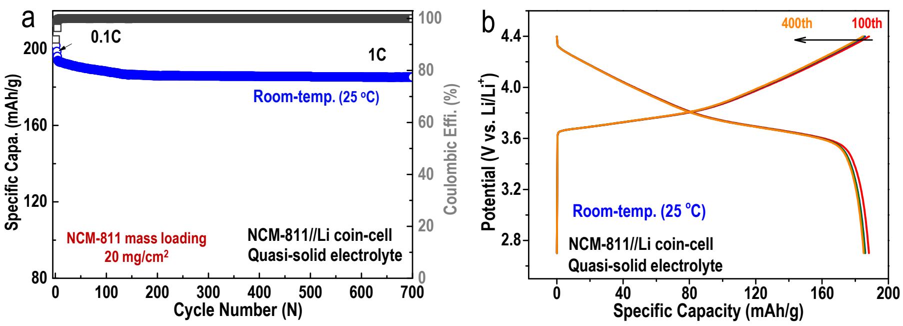

Figure S11. (a) cycling performance and the corresponding (b) discharge/charge curves of the NCM-811 coin-cell (NCM-811 cathode mass loading of about 20 mg cm^{-2}) used quasi-solid electrolyte under room-temperature (25 °C).

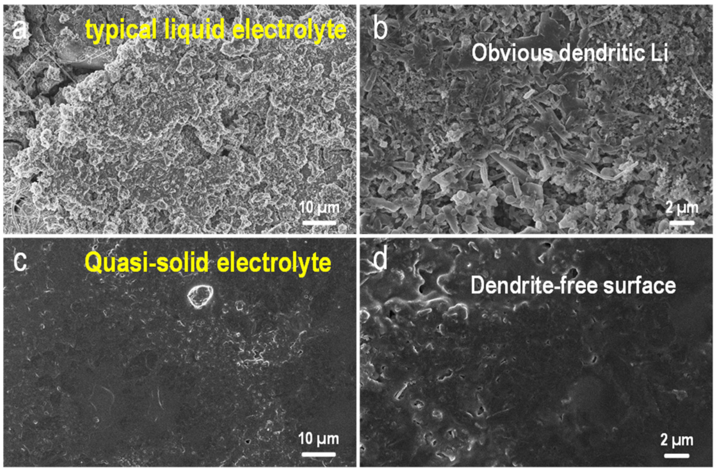

Figure S12. SEM of the cycled Li harvested from Li//Li cells assembled with (a, b) typical liquid electrolyte after 100 hours and (c, d) quasi-solid electrolyte after 600 hours.

Li anodes harvested from the cycled Li//Li cell used quasi-solid electrolyte demonstrated smooth surface and without dendritic Li can be observed.

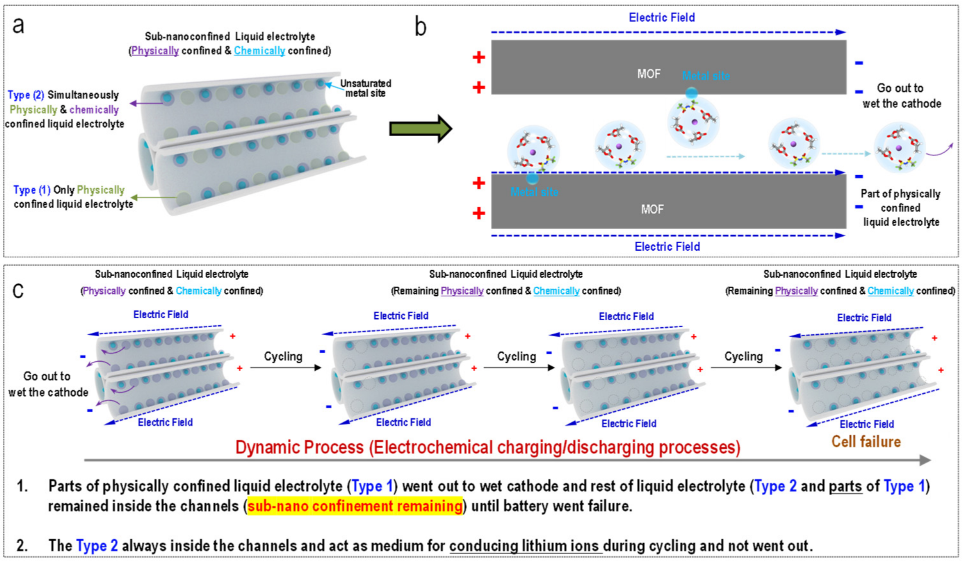

Figure S13. (a) Schematic illustration of two types of liquid electrolytes inside the MOF channels. (b) Schematic illustration of working mechanism for the MOF-based quasi-solid electrolyte in this work. (c) How the "sub-nano confinement" effect maintained during the electrochemical cycling processes.

As shown in Figure S13, it was thought for the prepared MOF-based quasi-solid electrolyte, there are two types of liquid electrolytes inside the MOF channels: Type 1 electrolyte: the physically confined liquid electrolyte inside MOF channels (solely confined inside the sub-nano channels of MOF) and Type 2 electrolyte: simultaneous physically & chemically confined liquid electrolyte (chemically coordinated by unsaturated Cu metal sites inside MOF channels while also physically confined inside the sub-nano channels). Therefore, as schematically demonstrated in Figure S13a, both Type 1 and Type 2 electrolyte were under the sub-nanoconfinement effect constructed by the narrow MOF channels. It was thought that only Type 1 electrolyte can go out and wet the cathode while the Type 2 electrolyte did not go out the MOF channels (as schematically illustrated in Figure S13b). The mechanism of how the "sub-nano confinement" effect maintained during the electrochemical cycling processes was also proposed and shown in Figure S13c. As demonstrated in Figure S13c, during cycling, especially at the initial stage, only very small amount of Type 1 electrolyte went out from the MOF channels to wet the NCM-811 cathode. As the electrochemical process continues, more Type 1 electrolyte go out the MOF channels This dynamic process will stop until all the Type 1 electrolyte that originally inside the MOF channels fully go out and be totally consumed. Therefore, during this dynamic process, there are remaining Type 1 and the unreduced/unchanged amount Type 2 electrolyte inside the MOF channels before all the Type 1 electrolyte is totally consumed. Thus, the sub-nanoconfinement will always exist during the multiple electrochemical cycling processes. More importantly, even if the Type 1 electrolyte is totally consumed, while the Type 2 electrolyte did not go out from the MOF channels (as schematically illustrated in Figure S13b), the sub-nano confinement effect will always exist.

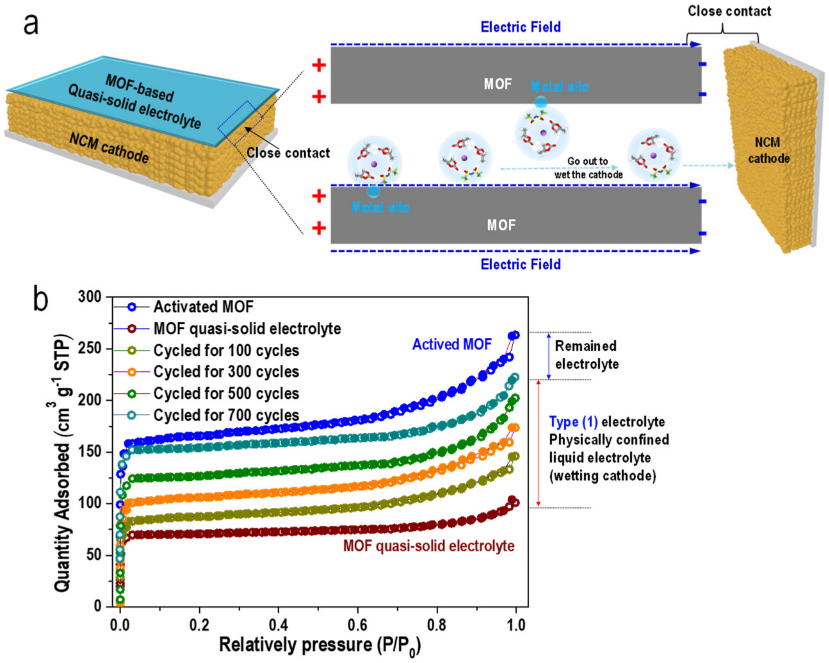

Figure S14. (a) Schematic illustration of working mechanism for the MOF-based quasi-solid electrolyte in this work. (b) BET curves of cycled MOF-based quasi-solid electrolytes after different cycles.

As shown in Figure S14a (similar as Figure S13b), only Type 1 electrolyte can go out and wet the cathode while the Type 2 electrolyte did not go out the MOF channels. Clearly, as shown in Figure S14b, the gap between activated MOF and the newly prepared MOF-based quasi-solid electrolyte represented the overall amount of electrolyte (both Type 1 and Type 2 electrolyte) confined inside MOF channels. After cycled for 100, 300 and 500 cycles, electrolyte inside MOF channels decreased gradually, which can be ascribed to the constantly consuming of Type 1 electrolyte. The gradually consumed electrolyte also further indicated during cycling, there were remaining Type 1 (and the unreduced/unchanged amount Type 2 electrolyte) inside the MOF channels before all the Type 1 electrolyte is totally consumed. For example, after cycled for 100 cycles, small amount of Type 1 electrolyte was consumed. However, there was still enough Type 1 electrolyte remained for the following 300, 500, and 700 cycles. The same conclusion can be made when cycled for 300 and 500 cycles. The gap between activated MOF and the MOF-based quasi-solid electrolyte after 700 cycles represented the remained electrolyte inside the MOF channels. Thus, the sub-nanoconfinement (only for Type 1 electrolyte) will always exist during the multiple electrochemical cycling processes. More importantly, even if the Type 1 electrolyte is totally consumed, while the Type 2 electrolyte did not go out from the MOF channels (indicated by the (111) peaks of different MOF-based quasi-solid electrolyte after cycling shown in Figure 5i), the sub-nano confinement effect will always exist.

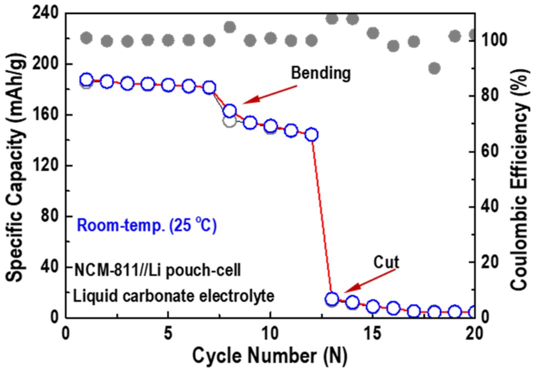

Figure S15. Cycling performances of NCM-811//Li pouch-cell (NCM-811 cathode mass loading about 20 mg cm^{-2}) assembled with quasi-solid electrolyte under room temperature (25 ºC) after being damaged (bended and cut).

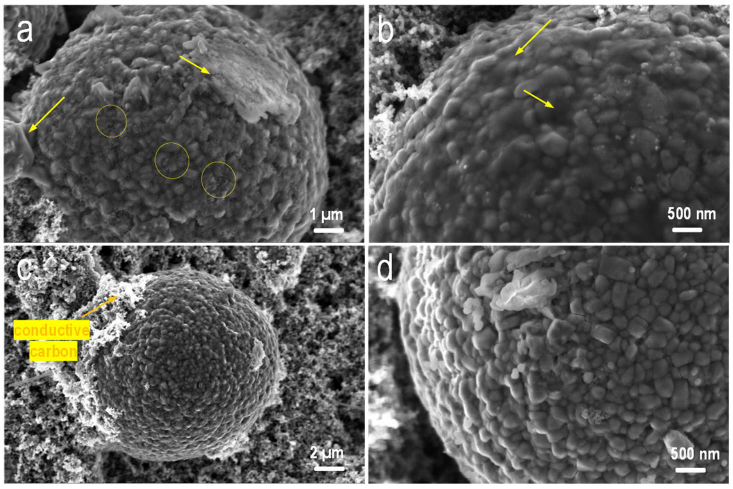

Figure S16. SEM images of the cycled NCM-811 cathodes from NCM-811//Li pouch-cells used (a, b) typical liquid electrolyte and (c, d) the prepared quasi-solid electrolyte that cycled under high temperature of 90 ºC (yellow curves demonstrated in Figure 5c and Figure 5e).

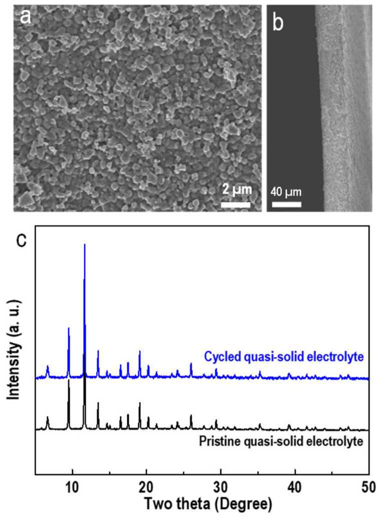

Figure S17. Stability of the cycled quasi-solid electrolyte. (a, b) SEM images of the cycled quasi-solid electrolyte. (c) XRD pattern of the cycled quasi-solid electrolyte.

Supplementary References

1. Chang, Z. et al. Beyond the concentrated electrolyte: further depleting solvent molecules within a Li+ solvation sheath to stabilize high-energy-density lithium metal batteries. *Energy & Environmental Science* **13**, 4122-4131 (2020).
2. Li, J.F. et al. Shell-isolated nanoparticle-enhanced Raman spectroscopy. *Nature* **464**, 392-395 (2010).
